# Overview of SARS-CoV-2 genomic surveillance in Central America and the Dominican Republic from February 2020 to January 2023: the impact of PAHO and COMISCA's collaborative efforts

**DOI:** 10.3389/fpubh.2026.1738843

**Published:** 2026-02-11

**Authors:** Sofía Herrera Agüero, Aldo Sosa, Alexander A. Martinez, Ambar Moreno, César Roberto Conde Pereira, Claudia Gonzalez, Claudio Soto-Garita, Daniel Ulate, Estela Cordero-Laurent, Hebleen Brenes, Isaac Miguel Sánchez, Jairo Mendez-Rico, Jessica Góndola, Jose Arturo Molina Mora, Juliana Leite, Leticia Franco, Linda Mendoza, Lionel Gresh, Lucia De La Cruz, Mitzi Castro Paz, Monica Barahona, Naomi Iihoshi, Oris Chavarría, Priscila Born, Ruby Melany Aguillón, Ruth Carolina Vasquez Cordova, Selene Gonzalez, Sofia Carolina Alvarado Silva, Xochitl Sandoval López, Yvonne Imbert, Francisco Duarte-Martínez

**Affiliations:** 1 Instituto Costarricense de Investigación y Enseñanza en Nutrición y Salud, Cartago, Costa Rica; 2 Centro de Investigación en Enfermedades Tropicales and Facultad de Microbiología, Universidad de Costa Rica, San José, Costa Rica; 3 Medical Laboratory Services, Ministry of Health and Wellness, Belmopan, Belize; 4 Department of Genomic and Proteomic, Gorgas Memorial Institute of Health Studies, Panama City, Panama; 5 National Research System (SNI), National Secretary of Research, Technology and Innovation (SENACYT), Panama City, Panama; 6 Department of Microbiology and Immunology, University of Panama, Panama City, Panama; 7 Laboratorio Nacional de Salud, Ministerio de Salud Pública y Asistencia Social, Villa Nueva, Guatemala; 8 Laboratorio Nacional de Salud Pública Dr. Defilló, Ministerio de Salud Pública y Asistencia Social, Santo Domingo, Dominican Republic; 9 Infectious Hazard Management Unit, Health Emergencies Department, Pan American Health Organization, Washington, DC, United States; 10 Laboratorio Nacional de Vigilancia de la Salud, Hospital San Felipe, Tegucigalpa, Honduras; 11 Secretaría Ejecutiva del Consejo de Ministros de Salud de Centroamérica y República Dominicana (SE-COMISCA)/Sistema de la Integración Centroamericana (SICA), San Salvador, El Salvador; 12 Instituto Nacional de Salud, Ministerio de Salud de El Salvador, San Salvador, El Salvador

**Keywords:** Central America, coronavirus, COVID-19, genomic surveillance, regional collaboration, SARS-CoV-2

## Abstract

This study provides a comprehensive overview of SARS-CoV-2 genomic surveillance in Central America and the Dominican Republic from February 2020 to January 2023, highlighting the collaborative efforts of the Pan American Health Organization (PAHO), and the Council of Ministers of Health of Central America (COMISCA). A total of 26,595 sequences from the GISAID database were analyzed, correlating findings with key events reported by participating entities. The genomic analysis reveals significant co-circulation of variants, with notable lineage diversity observed throughout the pandemic. Variants of concern (VOC) like Alpha, Gamma, Delta and Omicron were identified alongside variants of interest (VOI) like Lambda and Mu. The emergence of recombinant lineages further illustrates the ongoing evolution of the virus and its spread across the region, underscoring the interconnectedness of Central America and the Dominican Republic. The collaborative model facilitated broader sequencing coverage, enabling more extensive surveillance than individual countries could achieve alone. Despite the successes of regional collaborations, challenges remain, particularly regarding sequencing capacity in countries impacted by socioeconomic inequalities. Addressing these gaps is essential to enhance public health responses to current and future pandemics.

## 1 Introduction

In late 2019, numerous cases of pneumonia with an unknown etiology were detected in the city of Wuhan, China. The causal agent was identified as a novel type of coronavirus and was named SARS-CoV-2 (1). The World Health Organization (WHO) officially designated the illness caused by SARS-CoV-2 as COVID-19 (coronavirus disease 2019). Subsequently, in March 2020 this emerging outbreak was declared a global pandemic (1, 2).

As an RNA virus, SARS-CoV-2 undergoes frequent mutations as part of its replication cycle. The high replication rates during the pandemic resulted in the emergence of new genotypes (3). The emergence of variants that had likely a greater effect in public health led the WHO to categorize them based on shared attributes and required public health actions. These categories include variants of concern (VOC), variants of interest (VOI) and variants under monitoring (VUM) (4). Notably, variants that were initially classified as VOC, like Alpha, Beta, Gamma, Delta and Omicron, were all reported in Central America (5, 6). Additionally, several lineages were first observed in countries within Central America and the Dominican Republic (7–10). These findings underscore the critical importance of genomic surveillance in this region to monitor the spread and evolution of the virus effectively.

The COVID-19 pandemic emphasized the significance of genomic surveillance, shedding light on the challenges associated with its implementation in the various public health systems. Consequently, since the beginning of the pandemic, government entities have made substantial investments toward the advancement and implementation of whole genome sequencing (WGS) (11, 12).

The implementation of an integrated genomic surveillance system during the pandemic has proven to be crucial for public health understanding and decision-making. This system has been instrumental in monitoring the real-time evolution of SARS-CoV-2 through the open exchange of data and online platforms like GISAID (Global Initiative on Sharing All Influenza Data, https://gisaid.org) (11, 12). By identifying and categorizing the various lineages of circulating viral diversity, this system has enabled the early detection of emerging variants before their widespread dissemination. Moreover, this monitoring made possible the linking of cases to clusters, tracing the origins of outbreaks, and identifying both national and international transmission routes. The knowledge gained about the different mutations present in circulating viruses was essential for the development of vaccines, treatments and diagnostic tests, as well as monitoring the impact of control measures (11, 12).

In Central America and the Dominican Republic, the progress made in genomic surveillance was strengthened by an effective collaboration model between the Pan American Health Organization (PAHO) and the Council of Ministers of Health of Central America (COMISCA). This collaboration aimed at enhancing capacities in the various countries of Central America and the Dominican Republic during the COVID-19 pandemic.

Thus, this study aimed to highlight the milestones in the strengthening of the genomic surveillance systems in the sub regions and to conduct a descriptive analysis of the lineages and circulating variants in Central America and the Dominican Republic from February 2020 to January 2023. This analysis is based on diverse SARS-CoV-2 genomes available in the GISAID database, sourced from material sequenced and shared by the public health laboratories within the subregion.

It is important to note that, although genomic and epidemiological data were jointly analyzed, the available metadata across countries did not consistently include key determinants such as vaccination coverage, prior infections, or the implementation and stringency of non-pharmaceutical interventions. As a result, statistical models assessing associations between circulating variants and reported cases or deaths could not incorporate these factors as covariates. This limitation inherently restricts the inferential scope of the models and should be considered when interpreting trends derived from the available data.

## 2 Materials and methods

### 2.1 Data acquisition

In order to conduct a comprehensive analysis of SARS-CoV-2 genomic surveillance in Central America and the Dominican Republic, datasets containing whole genome sequences and accompanying metadata were acquired from the GISAID repository. Data retrieval was performed on March 20th, 2023, and consisted of sequences voluntarily shared by participating countries. All genome sequences and associated metadata used in this study are accessible through the persistent digital object identifier https://doi.org/10.55876/gis8.251216zx, which aggregates the corresponding GISAID accession numbers and contributor information via the associated EPI_SET_251216zx identifier.

### 2.2 Timeline of key events

Key events leading to enhancing capacities in the various countries of Central America and the Dominican Republic during the COVID-19 pandemic were provided by PAHO (https://www.paho.org/en), the COMISCA Executive Secretary (SE-COMISCA; https://www.sica.int/comisca/inicio), Instituto Costarricense de Investigación y Enseñanza en Nutrición y Salud (INCIENSA; https://www.inciensa.sa.cr) and Instituto Conmemorativo Gorgas de Estudios de la Salud (ICGES; https://www.gorgas.gob.pa).

The timeline plot was generated using ggplot2 v3.4.4 R package. The complete R script is publicly accessible in GitHub (https://github.com/sofiaherreraa/SARS-CoV-2_CADR).

### 2.3 COVID-19 epidemiological data

COVID-19 case numbers and total deaths for each country were obtained from daily reports provided by Our World in Data (13). Sequenced SARS-CoV-2 samples for each country were sourced from the GISAID platform (https://gisaid.org), with data collection limited to samples collected up until January 31st, 2023.

A dataset was constructed comprising the monthly proportions of SARS-CoV-2 lineages, total reported cases and deaths, and a temporal index for analysis. These models were implemented to explore whether changes in dominant lineage proportions were associated with temporal patterns in reported cases and deaths. To reduce noise from lineages with minimal fluctuation, the variance of each lineage's proportion over time was calculated, and only those with a variance greater than 0.001 were retained. Two regression models were then implemented to assess the association between lineage proportions and epidemiological outcomes: a Poisson regression model for monthly case counts and another for death counts, each incorporating time and the selected lineage proportions as predictors. Overdispersion was assessed by calculating the ratio of the sum of squared Pearson residuals to the residual degrees of freedom. If this ratio exceeded 1.5, a negative binomial regression was used instead of Poisson regression to account for extra variability. All model coefficients, standard errors, z-values, and *p*-values are presented in Supplementary Table S2. Normalized case/death counts were scaled using min–max normalization (0–1 range). The secondary axis represents the normalized values, aligned to facilitate visual comparison with lineage proportions.

The models in this study rely exclusively on reported cases, deaths, and lineage prevalence. Due to the absence of consistent metadata across all countries—such as vaccination rollout timing, NPIs, prior infection levels, or testing access—these contextual factors could not be incorporated. These limitations, together with differences in reporting quality among countries, should be considered when interpreting trends presented in this study.

Additionally, a standardized regional case definition or testing strategy was not implemented across the participating countries. Each national public-health authority maintained its own surveillance and diagnostic framework, resulting in differences in testing access, case ascertainment, and sampling workflows. Sample selection for sequencing followed country-specific laboratory protocols and resource availability, generally prioritizing routine surveillance samples or those designated by national programs. Consequently, sequencing representativeness varies between countries and does not necessarily reflect population-level sampling.

The interval between sample collection and deposition in GISAID was not analyzed as a proxy for sequencing turn-around times due to heterogeneous metadata and differences in national and regional workflows. In several settings, samples were stored frozen for extended periods before sequencing or shipment to external laboratories, which—together with inconsistent reporting—could introduce biases and not accurately reflect sequencing capacity or public health responsiveness.

Lineage assignments were not independently generated for this study. Instead, the Pango lineage calls provided directly in the GISAID metadata were used as reported at the time of data download.

All statistical analyses and visualizations, including descriptive trends, regression models, and the generation of Supplementary Table S2, were performed using R (version 3.4.4), primarily with the ggplot2 package. The complete R script is publicly accessible in GitHub (https://github.com/sofiaherreraa/SARS-CoV-2_CADR).

### 2.4 Analysis of circulating variants

To assess the enhancing capacities in the various countries of Central America and the Dominican Republic during the COVID-19 pandemic throughout the period of study, genomic sequences deposited in GISAID from each country were analyzed longitudinally. The initial dataset comprised a total of 26,595 sequences, covering the period from February 2020 to January 2023. These sequences were sourced from eight countries (COMISCA members) within the region, specifically: Costa Rica, Panama, Guatemala, Dominican Republic, Belize, Nicaragua, El Salvador, and Honduras.

Pango lineage designations obtained from the GISAID were used to analyze the lineage frequency across countries (10, 14). The analysis focused on identifying and characterizing the predominant lineages circulating in these regions. Specifically, the top 14 most prevalent lineages were individually assessed. Additional exploratory analyses were conducted using the top 30, 50, and 100 lineages (Supplementary Figure S1); however, these broader selections did not provide added interpretive value and introduced considerable noise, leading to a focus on the 14 most representative ones for clarity and relevance.

To further investigate the relevance of these lineages within the context of Central America and the Dominican Republic, an overall threshold of 35 sequences (*n* > 35) per month was applied across the full regional dataset to determine which lineages were assessed individually. Lineages not meeting this threshold were grouped under the category “others.” This cutoff was selected based on internal comparisons of different thresholds (*n* = 35, 50, 75, and 100; data not shown) to optimize clarity and ensure representativeness. Lower thresholds led to the inclusion of many low-frequency lineages with limited interpretive value, while higher thresholds excluded lineages that were relevant in some months but not consistently abundant. The final set of lineages identified through this overall threshold was then used consistently across the individual country panels. Any lineages not included in the overall panel were grouped as “others” in the country-specific figures to maintain comparability and minimize noise.

All plots were generated using ggplot2 v3.4.4 R package. The complete R scripts are publicly accessible on GitHub (https://github.com/sofiaherreraa/SARS-CoV-2_CADR).

### 2.5 Phylogenetic analysis

Sequences were accessed on March 20th 2023. The official GISAID Author Acknowledgment Table corresponding to this dataset has been included as Supplementary Table S1. Sequences were selected based on a minimum coverage threshold of 28,000 (approximately 95% of the full SARS-CoV-2 genome length) and less than 6% ambiguous bases (Ns; *N*), to ensure high-quality assemblies and minimize biases associated with incomplete or low-confidence genomes. This filtering yielded a dataset of 25,185 sequences.

To reduce computational load while maintaining representative temporal coverage, a stratified sampling was then conducted annually from February 2020 to January 2023, using Subsampler v1.1.0 (15). This sampling approach resulted in a total of 2,487 sequences, equivalent to an average sampling rate of 9.875% per year.

Sequence alignment was performed using MAFFT v7.471 (16), followed by a maximum likelihood-based phylogenetic analysis with IQ-TREE v1.6.12 (17), employing the GTR+F+R2 substitution model selected by ModelFinder (18). The resulting phylogenetic tree was visualized using iTOL v4 (19), incorporating metadata.

## 3 Results

### 3.1 Implementation of genomic surveillance and sequencing capacity

**Figure 1:**
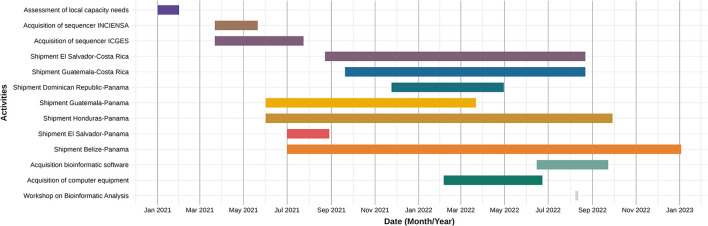
Timeline of processes implemented to strengthen genomic surveillance of SARS-CoV-2 in Central America and the Dominican Republic.

From February 2020 to January 2023, genomic surveillance efforts in Central America and the Dominican Republic identified multiple SARS-CoV-2 lineages and variants. A timeline of key events is illustrated in Figure 1, to highlight the processes implemented to enhance genomic surveillance capacities in the various countries of Central America and the Dominican Republic during the COVID-19 pandemic. In January 2021, the local laboratory capabilities in the selected countries, INCIENSA in Costa Rica and ICGES in Panama, were assessed. Based on the assessment, it was identified that INCIENSA and ICGES required additional sequencing equipment, supplies, reagents and staff to support the laboratories (20, 21). Additionally, during this process, REDLAB (the Network of National Public Health Laboratories of Central America and the Dominican Republic) reached an agreement with its laboratory members to participate in this activity, which involved sending out SARS-CoV-2 positive samples for sequencing. As seen in Figure 1, shipment of these samples began in February 2021 up until January 2023, where some countries shipped to Panama (ICGES) and others to Costa Rica (INCIENSA).

To describe the SARS-CoV-2 genomic surveillance in Central America and the Dominican Republic, Table 1 presents statistical COVID-19 data from the beginning of the pandemic up until January 31st, 2023. Nicaragua shows the highest percentage of sequenced COVID-19 cases; however, this data may be biased due to the country's surveillance strategies. Therefore, it will not be considered for comparative analyses. Belize also shows a relatively higher percentage of sequenced COVID-19 cases compared to other evaluated countries. This discrepancy is likely influenced by Belize's relatively small population of approximately 400 thousand inhabitants (22).

**Table 1:** COVID-19 statistical data in Central America and the Dominican Republic from the beginning of the pandemic to January 31st, 2023.

**Country**	**COVID-19 cases per million**	**Total deaths**	**Total reported COVID-19 cases**	**Total sequenced SARS-CoV-2 samples**	**Percentage of sequenced COVID-19 cases (%)**	Genomes retrieved from GISAID^*^	Completeness average (%)^*^	Sequences with ≥95% coverage and < 6% ambiguities^*^	Sequences that met the quality criteria (%)^*^
Belize	174,223.1	688	70,610	1,183	1.68	1,071	99.37	970	90.57
Costa Rica	228,954.6	9,158	1,186,176	9,960	0.84	9,921	99.35	9,865	99.44
Dominican Republic	58,793.97	4,384	660,187	2,628	0.40	2,585	99.46	2,345	90.72
El Salvador	31,845.41	4,230	201,785	914	0.45	633	99.48	616	97.31
Guatemala	68,736.55	20,092	1,226,529	4,623	0.38	4,504	99.32	4,053	89.99
Honduras	45,103.27	11,104	470,556	287	0.06	233	99.43	225	96.57
Nicaragua	2,240.661	245	15,569	1,065	6.84	1,064	98.79	768	72.18
Panama	233,567.4	8,596	1,029,701	6,613	0.64	6,584	99.28	6,343	96.34

Values marked with an asterisk (^*^) refer to sequences collected within the time window from February 1st, 2020 to January 31st, 2023.

To ensure quality in downstream analyses, retrieved sequences were filtered by completeness and ambiguity thresholds. In most countries, over 90% of the sequences met the quality criteria (≥95% genome coverage and < 6% ambiguous bases), except for Nicaragua, where only 72% of sequences passed the filters, potentially reflecting differences in sequencing quality or protocols.

Costa Rica and Panama have higher percentage of sequenced COVID-19 cases (0.84 and 0.64%) compared to the other countries in the region. Conversely, Honduras has a significantly lower percentage of sequenced cases at 0.06%. The Dominican Republic, El Salvador and Guatemala have similar percentages and are slightly lower than those reported for Costa Rica and Panama.

Periods without sample shipments should not be interpreted as definitive gaps in genomic surveillance. The shipment records captured only the samples processed through PAHO/COMISCA-supported pipelines, and the available documentation does not allow determination of whether countries continued sequencing or conducted other forms of viral characterization, such as lineage assignment or partial typing, through alternative mechanisms during those intervals. Therefore, shipment gaps reflect only the absence of documented transfers and not necessarily the absence of genomic surveillance activities.

### 3.2 Circulating variants and lineage

**Figure 2:**
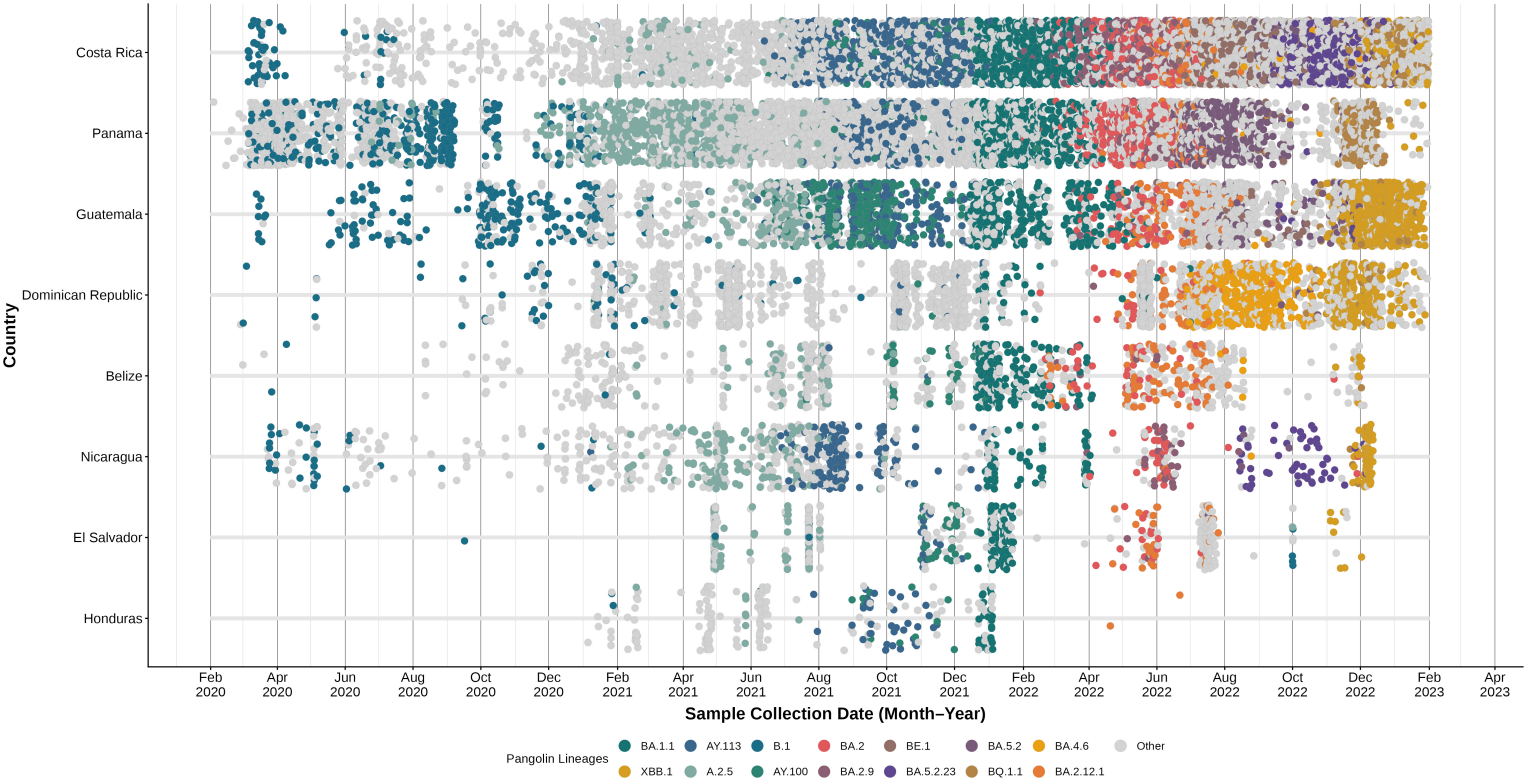
Individual SARS-CoV-2 sequences obtained by country in Central America and Dominican Republic from February 2020 to January 2023. The top 14 most prevalent lineages were individually labeled, and all the remaining lineages were labeled as other.

The data illustrated in Figure 2 highlights the distribution of circulating SARS-CoV-2 variants over time in each country. At the start of the pandemic, the Dominican Republic, Belize, El Salvador, and Honduras had minimal to no sequenced SARS-CoV-2 cases. A noticeable increase in sequencing activity was observed from early 2021 onward.

Figure 2 displays that the same SARS-CoV-2 lineages were detected across several countries during overlapping time periods. Early in the pandemic, despite limited sequencing in some countries, the B.1 lineage was reported throughout the region. In mid-2021, Delta sub-lineages AY100 and AY113 (23) were present across the included countries. The Omicron sub-variant BA.1.1 (24) was identified in samples from Central America and the Dominican Republic between December 2021 and January 2022.

**Figure 3:**
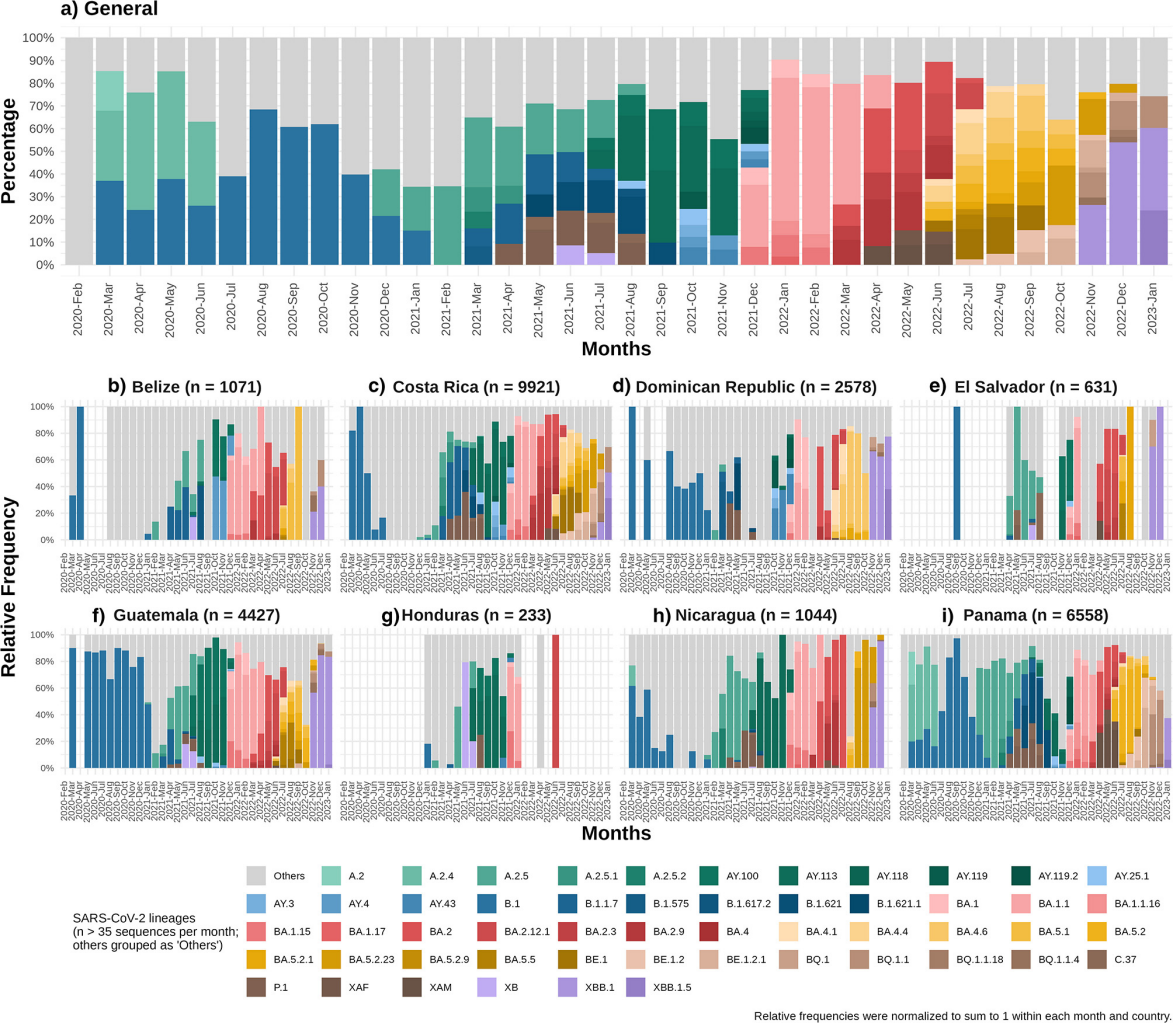
Monthly relative frequency of SARS-CoV-2 lineages circulating in Central America and the Dominican Republic from February 2020 to January 2023. **(a)** Overall lineage distribution in the region. Lineages with a cumulative frequency greater than 35 sequences were shown individually; less frequent lineages (*n* ≤ 35) were grouped under “Others.” Percentages represent the relative frequency of each lineage per month. **(b–i)** Country-specific lineage distributions. The lower panels display the monthly distribution of lineages in each country. Only months with available data are shown, with blank spaces indicating the absence of sampling. The color palette and lineage grouping are consistent with the overall chart, including the “Others” category.

To illustrate the temporal dynamics of circulating SARS-CoV-2 lineages in Central America and the Dominican Republic, Figure 3 presents a composite area plot showing the monthly proportion of lineages at the regional level and in each country. The number of lineages included was filtered using an overall threshold of more than 35 sequences, as lower-frequency lineages provided limited additional interpretive value. The same color palette and lineage classification used in the regional summary were applied to the country-level plots to ensure consistency in interpretation.

As shown in Figure 3, no lineage exceeded 35 occurrences in February 2020. Between March 2020 and February 2021, at least one lineage consistently surpassed this threshold, representing approximately 20% of sequenced samples each month.

Between March 2021 and December 2021, the number of lineages exceeding 35 occurrences increased significantly. By September 2021, Delta sublineages AY.100 and AY.113 comprised more than 50% of the circulating lineages; with Delta's dominance maintained in October and November 2021 with the rise of new sublineages (23). However, in January, February, and March 2022, lineage diversity with more than 35 occurrences was significantly reduced due to the dominance of the Omicron subvariant BA.1.1 (24), which had been prevalent since December 2021, and accounted for at least 50% of cases during the first 3 months of 2022. From April 2022 to November 2022, lineage diversity exceeding 35 occurrences rose again. In December 2022, the recombinant lineage XBB.1 (25) emerged, comprising more than 50%, resulting in a decline in overall lineage diversity. By January 2023, three Omicron lineages predominated: XBB.1, XBB.1.5 and BQ1.1, with the first two being recombinant lineages (25).

### 3.3 Phylogenetic analysis of SARS-CoV-2 lineages

**Figure 4:**
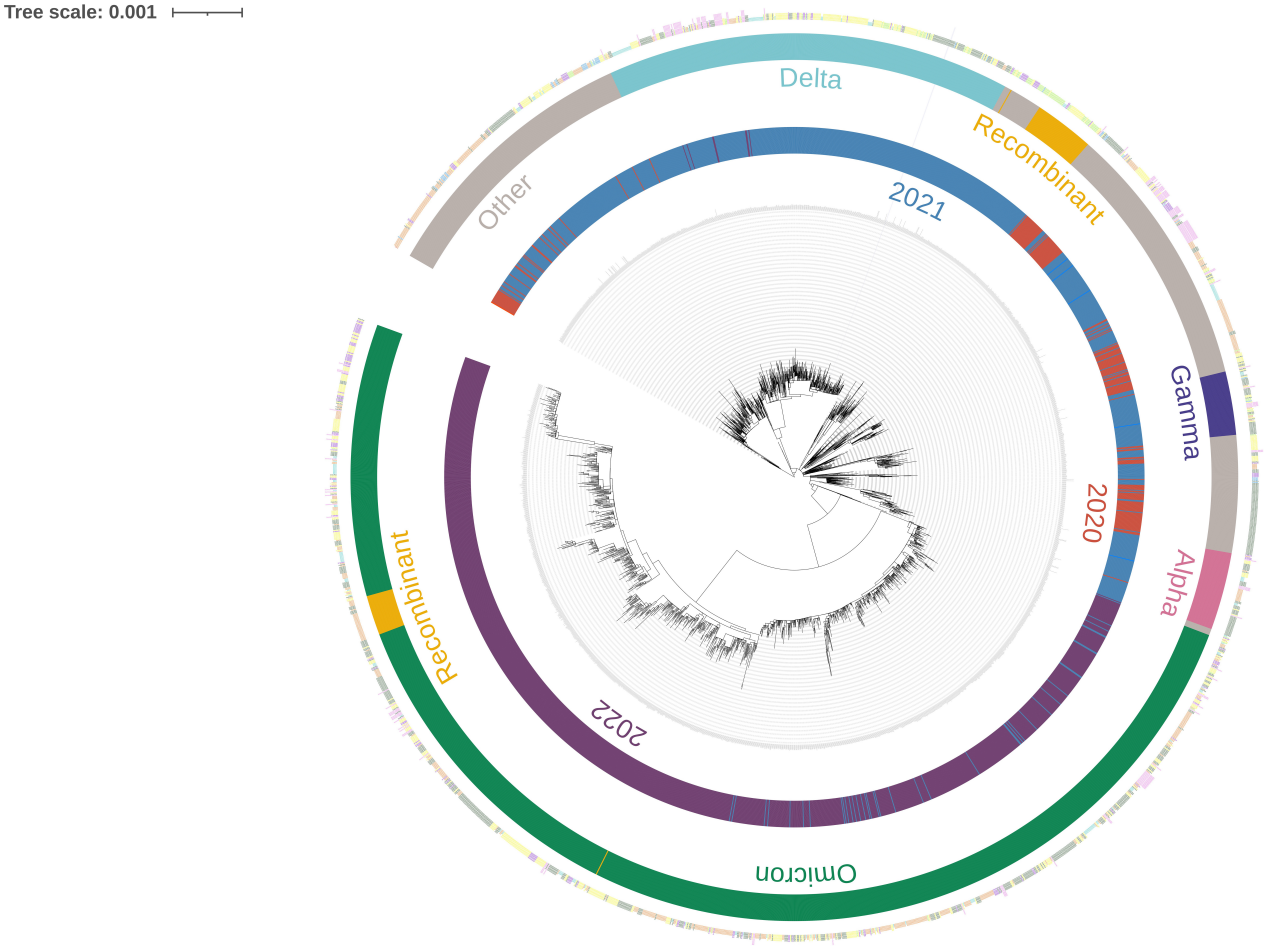
Maximum likelihood phylogenetic tree of SARS-CoV-2 sequences from Central America and the Dominican Republic, with temporal and geographic annotations, spanning February 2020 to January 2023.

Figure 4 illustrates the evolution of different variants throughout the study period in Central America and the Dominican Republic, presenting temporal and country-specific information. In 2020, no clear predominance of any variant was observed; however, variants such as Gamma and Alpha, along with closely related lineages, were present to some extent. By 2021, Gamma, Alpha and Delta were present in significant proportions, with Delta showing a clear predominance. Omicron was also present to a lesser extent. In 2022, Omicron became the dominant variant, while Delta was present in smaller amounts.

### 3.4 Associations between lineage dynamics and epidemiological trends

**Figure 5:**
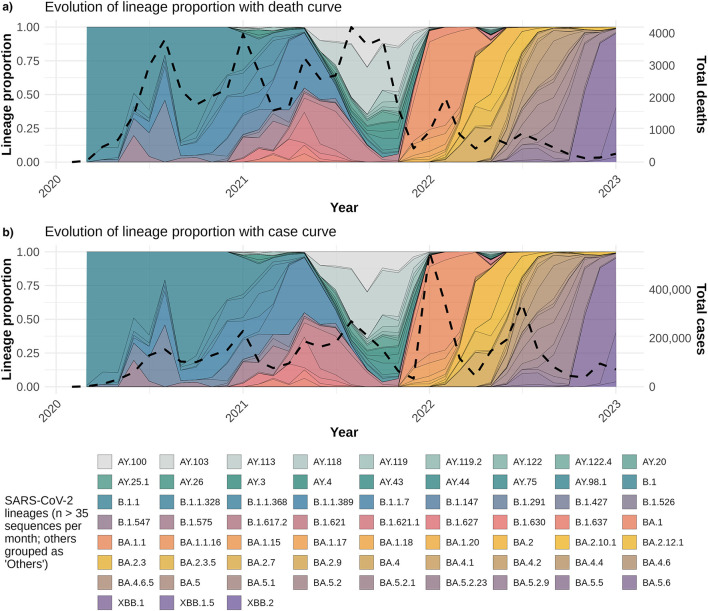
Temporal dynamics of SARS-CoV-2 lineage proportions with COVID-19 statistical curves in Central America and the Dominican Republic. **(a)** Monthly proportions of SARS-CoV-2 lineages detected by genomic surveillance are shown as stacked areas. The black dashed line represents the normalized total monthly COVID-19 deaths (secondary *y*-axis), facilitating comparison between lineage prevalence and mortality trends over time. **(b)** Same as **(a)**, but the black dashed line depicts the normalized total monthly reported COVID-19 cases instead of deaths. Lineage proportions are calculated as the fraction of total cases sequenced each month. Colors correspond to specific viral lineages.

Figure 5 overlays lineage proportions with normalized monthly COVID-19 cases (panel B) and deaths (panel A). Cases and deaths were normalized using min–max scaling (0–1 range) and plotted on a secondary *y*-axis to facilitate comparison with lineage prevalence trends. From 2022 onwards, certain lineages such as BA.1.1, BA.2, and their sublineages showed marked increases in prevalence concurrent with rises in reported case numbers. Conversely, the monthly death counts exhibited a downward trend during the same period. Negative binomial regression models were fitted to assess associations between lineage proportions and epidemiological metrics. While a few lineages demonstrated statistically significant associations—such as BA.2.9 with case counts (*p* = 0.028) and XBB.1 with death counts (*p* = 0.015)—the overall explanatory power of individual lineages was limited. Full regression outputs, including coefficients and significance values, are provided in Supplementary Table S2.

## 4 Discussion

Over the years, outbreaks like severe acute respiratory syndrome (SARS; 2002–2023), influenza (2009) and Ebola (2013–2014) have highlighted the need for genomic surveillance to support public health surveillance systems (26). During the COVID-19 pandemic, genomics played a key role by enabling the tracking of SARS-CoV-2 variants and their spread, supporting the development of molecular diagnostics techniques, therapeutics and vaccines, fand informing decisions on the strengthening or easing of social distancing measures (27). This study did not assess whether genomic surveillance outputs directly impacted policy decisions, nor did it evaluate the timeliness of sequence generation and sharing. Turnaround times differed across countries, especially where external sequencing support was required, which may have limited the use of genomic data for real-time public health responses.

Interpretation of lineage frequencies must consider potential selection biases, as sequencing strategies were not systematic across countries. Differences in testing access, diagnostic algorithms, and sample triaging procedures likely influenced which samples were ultimately sequenced. These factors limit the comparability of lineage proportions across settings and should be considered when interpreting regional trends.

The recognition of the importance of genomic surveillance during the COVID-19 pandemic led to organizations such as PAHO (20) and COMISCA (21) to enhance genomic surveillance efforts across Central America and the Dominican Republic. A key objective of these strengthening programs was the development of local bioinformatics analysis capacity. As of 2025, many countries are still in the early stages of developing these skills.

In this context, INCIENSA in Costa Rica and ICGES in Panama were key players, as they led the implementation of SARS-CoV-2 genomic sequencing, as illustrated in Figure 1 (21). These efforts led to enhanced sequencing capacities among Central America and the Dominican Republic. As demonstrated in Figure 2, several countries with little to no sequenced samples throughout 2020 experienced a significant increase in sequencing activities in 2021, with many sustaining this progress into 2022.

As illustrated in Table 1, Nicaragua and Belize exhibit the highest percentages of sequenced COVID-19 cases. Nevertheless, as previously mentioned, Nicaragua's data may be biased due to national surveillance strategies and was therefore not considered for comparative purposes.

In contrast, Belize's elevated percentage of sequenced COVID-19 cases can be attributed to its substantially smaller population compared to the other countries analyzed in this study. Belize's population is approximately 11 times smaller than that of Panama, the country with the next smallest population in the region (22).

After excluding these outliers, a clearer assessment of the sequencing capacity in Central America and the Dominican Republic can be achieved. Table 1 shows that Costa Rica and Panama had higher percentages of sequenced COVID-19 cases (0.84 and 0.64%, respectively) than other countries in the region. This is expected, as both countries led regional sequencing efforts and had established local access to equipment and reagents (21). In contrast, the Dominican Republic, El Salvador, Guatemala and Honduras relied on shipment of samples to Panama or Costa Rica for sequencing (Figure 1), contributing to their lower percentages. Honduras showed particularly low coverage (0.06%), which may be associated with limited testing capacity, exacerbated by Hurricanes Eta and Iota and a concurrent dengue epidemic (28).

Previous studies suggest that sequencing at least 5% of all positive tests allows the detection of emerging strains with prevalence between 0.1 and 1.0% (29). However, none of the countries achieved this threshold (Table 1). Globally, only 6.8% of the countries worldwide achieved sequencing coverage ≥5%, while 45.5% sequenced less than 0.5% of confirmed cases (30). The low coverage observed in Central America and the Dominican Republic therefore reflects a broader global trend, particularly among low- and middle-income countries. This underscores the global disparities in SARS-CoV-2 genomic surveillance (30). Although sequencing coverage remained below the recommended 5% threshold (29) in the region, the genomic surveillance efforts still allowed the detection of circulating VOCs and recombinant lineages (Figures 3, 4). Lower coverage likely reduced sensitivity for early detention of novel variants. Sustained investment in sequencing capacity, standardized sampling, and improved turnaround times would strengthen future surveillance efforts.

Despite limited sequencing volume, the quality of the sequences was generally high. Across countries, the average completeness exceeded 98%, and most sequences met the quality threshold of ≥95% coverage and < 6% ambiguities. Although Nicaragua showed a lower proportion of high-quality sequences (72.2%), all other countries consistently surpassed 90%, indicating robust technical performance despite limited resources.

Understanding the genomic landscape of SARS-CoV-2 is essential for tracking its evolution and informing public health responses. Analysis of genotypes and their circulation patterns provides insights into how different variants emerge and spread within populations. A notable finding was the synchronous circulation of multiple lineages across the region, reflecting active dynamic transmission. Similar dynamics have been documented in Latin American countries (31–33), other continents (34, 35) and globally, largely facilitated by international travel as part of modern globalization (36).

These patters highlight the importance of robust communication and coordination between neighboring countries and those with strong travel and trade connections. Effective monitoring of circulating strains requires cross-border collaboration, particularly in highly interconnected regions. The frequency of different lineages over time and their geographic distribution, can be observed in Figure 2. In early stages of the pandemic, the B.1 lineage was detected throughout Central America and Dominican Republic. By early 2021, A 2.5 also emerged across these countries. Mid- 2021, saw the rise of Delta sublineages AY.100 and AY.113 (23), while early 2022, marked the widespread presence of Omicron sublineages, with BA.1.1 (2), becoming the first predominant sub lineage. This omicron trend continued through 2022 and early 2023, with recombinant Omicron sublineages such as XBB.1 (25), becoming prominent by late 2022 and early 2023. The synchronous presence of recombinant lineages, such as XBB.1, further underscores the regional interconnectedness and transmission dynamics, highlighting the need to have capacity to detect the locally generated new variants.

The temporal dynamics of lineage circulation in Central America and the Dominican Republic differed from global trends. Unlike in Europe and North America, where Alpha rapidly became dominant, in this region Alpha (B.1.1.7) did not reach predominance (37, 38). Its spread was constrained by the concurrent circulation of Gamma (P.1) (39), Mu (B.1.621) (40), and Lambda (C.37) (41), which were already established locally and likely competed for transmission advantage. These dynamics illustrate how regional epidemiological conditions can modulate the replacement patterns seen globally.

Throughout 2020, the predominant lineages were A.2, A.2.4 and B.1, the only variants exceeding 35 cases until December 2020 (Figures 3, 4). Although Alpha was first detected in late 2020 (37), it remained limited during early 2021. By mid-2021, Gamma (P.1) emerged as a predominant variant (39), accompanied by the co-circulation of Lambda (C.37) and Mu (B.1.621), both classified as VOIs (40, 41). This produced a more diverse variant landscape compared to other regions.

Delta sublineages (VOC) became predominant by mid-2021, rapidly replacing Gamma and other variants, following global patterns (42). Among Delta, AY.100 and AY.113 were the most frequent sublineages in late 2021, peaking at ~60% of sequenced cases, while additional AY lineages such as AY.25.1, B.1.617.2, AY.119, AY.3, AY.4, and AY.43 contributed to transient diversification (23, 37). A similar pattern was observed in Mexico (2).

By December 2021, Omicron represented ~40% of regional cases with BA.1.1 as the most frequent subvariant (24), increasing to ~90% by January 2022, consistent with global displacement of Delta (43–46). Early 2022 showed reduced lineage diversity (Figure 3), which later increased as multiple Omicron sublineages co-circulated.

Recombinant lineages also contributed to regional dynamics. XB—parented by B.1.634 and B.1.631—was the first recombinant in the region to exceed 35 cases, peaking mid-2021 (7, 47). XAM (BA.1.1/BA.2.9) (48) and XAF (BA.1/BA.2) (7) were most prevalent in the second quarter of 2022, with XAF showing notable presence in Panama. By late 2022 and early 2023, XBB.1 and XBB.1.5 became predominant, accounting for >50% of cases, consistent with their global emergence patterns (49–51).

Overall, these observations highlight that variant dynamics in the region were shaped by both global introductions and strong competition among locally circulating VOCs and VOIs, particularly during the Alpha-to-Gamma/Mu period. This emphasizes the importance of considering regional epidemiological contexts when interpreting global variant trajectories.

The trends illustrated in Figure 5 reveal a noticeable decoupling between the temporal dynamics of SARS-CoV-2 lineages and epidemiological outcomes. The introduction and rapid expansion of Omicron sublineages (e.g., BA.1.1, BA.2, XBB.1.5) in early 2022 coincided with a marked increase in reported case numbers (Figure 5b), whereas COVID-19-related deaths continued to decline steadily during the same period (Figure 5a). This divergence was supported by a low overall correlation (*r* = 0.10) between the average monthly lineage proportions and total deaths. Additionally, although regression models using negative binomial distributions identified weak but statistically significant associations—such as BA.2.9 with decreased case counts (*p* = 0.028) and XBB.1 with reduced mortality (*p* = 0.0148)—no individual lineage accounted for a substantial portion of the variability in either trend (Supplementary Table S2). These patterns suggest that factors beyond lineage predominance, such as increased vaccination coverage, accumulated population immunity, or changes in clinical management and public health strategies, likely had a more prominent role in shaping the epidemic curve during the Omicron-dominant period. Therefore, these associations should be interpreted with caution, as the models did not incorporate vaccination, prior infections, NPIs, or other contextual factors.

The partnership between PAHO, COMISCA and the participant countries in Central America and the Dominican Republic was key to building regional capacity. It enabled countries without laboratories to access genomic surveillance and participate in sequencing efforts. This combined effort fosters the democratization of genomic tools in low- and middle-income countries, enabling them to contribute with data that supports evidence-based decision-making both at regional and global levels.

However, challenges remain, particularly in sequencing capacity in countries like Honduras impacted by socioeconomic inequalities and limited access to public health systems (30). Addressing these gaps is essential to ensure robust responses to future pandemics or outbreaks. Additionally, several countries presented prolonged gaps in sequencing activity, particularly during the early stages of the pandemic. These gaps may be attributed to logistical constraints, limited laboratory infrastructure, lack of trained personnel, or resource limitations in public health systems. For example, Honduras and El Salvador reported very few sequences during key periods (28). These disparities not only reflect structural inequalities across the region but also limit the representativeness of regional genomic trends. Similar gaps have been reported globally, approximately 63% of SARS-CoV-2 sequences lacked age or sex information and over 95% lacked clinical data such as vaccination status or symptom history (52).

Furthermore, genomic surveillance data plays a crucial role in informing public health decisions, including the development of appropriate tests, vaccines and treatment strategies (30). This highlights the necessity for continued regional cooperation to effectively manage viral evolution and expand surveillance efforts to encompass other pathogens and antimicrobial resistance, which has seen a notable rise in recent years (30, 53). Investing in genomic surveillance will enhance our understanding of viral circulation dynamics and health systems impact. It also strengthens infectious disease control strategies and supports real-time intervention management (53). Sustained genomic surveillance is vital for preparedness against future health threats, emphasizing the need for ongoing collaboration among countries.

Vaccination efforts across Central America and the Dominican Republic may have influenced the dynamics of lineage circulation (6). Although this study did not assess vaccination coverage by country, it is plausible that higher vaccination rates helped reduce the spread or persistence of certain variants. Conversely, delays in vaccine rollout could have provided favorable conditions for the expansion of specific lineages. Future analyses integrating vaccination timelines and coverage data with genomic trends would provide valuable insights into the relationship between immunization and variant dynamics (54).

The genomic surveillance data obtained during the study period supported public health decision-making processes in the region. For instance, the detection of specific lineages contributed to the revision of travel advisories and the adjustment of entry requirements in some countries (36). Additionally, the sequencing results provided evidence to refine vaccination strategies and prioritize populations at higher risk (11). Although the short-term efforts were successful, a long-term surveillance strategy is essential. This includes sustained funding, regional laboratory networks, workforce training, and integration of genomic data into routine public health response systems (26, 30).

Regional mobility likely played a critical role in the introduction and spread of SARS-CoV-2 lineages (36). While the study did not include travel history metadata, the emergence of similar lineages in neighboring countries within a short time frame suggests transboundary movement of the virus (31). Countries with stronger international connectivity, such as Panama and Costa Rica, may have served as primary entry points for founder lineages (55). Additionally, the lack of detected VOCs, VOIs or VUMs in some countries may reflect gaps in sequencing coverage rather than true absence of circulation (30). This underscores the need to integrate mobility data and improve coordinated genomic surveillance across borders.

Moreover, irregular migration may have contributed to viral spread through unconventional routes. One such situation is the Darién Gap, a dense jungle corridor between South America and Panama. In 2022, the Panamanian government reported over 250,000 migrants crossing this area under precarious conditions, potentially increasing the region's epidemiological connectivity (56). In addition, seasonal and binational migration among Indigenous populations, such as the Ngäbe–Buglé communities residing along the Panama–Costa Rica border, may also play a role in regional disease dynamics. These communities frequently migrate for agricultural work, particularly during Costa Rica's coffee harvest, and often settle in towns near production areas. Many Ngäbe individuals are binational citizens who regularly access health services in Costa Rica, following routes that overlap with known northbound migratory pathways. These movements underscore the need for integrated, cross-border genomic surveillance strategies that account for the health vulnerabilities of highly mobile and underserved populations (57). However, this interpretation remains speculative, as the available data do not allow determination of whether mobility in these population groups had any measurable or disproportionate effect on the observed variant dynamics.

While several genomic surveillance studies have been conducted in Latin America (58–60), to our knowledge, this is the first study to describe the regional characteristics of the SARS-CoV-2 genome in Central America and the Dominican Republic. These findings provide critical public health insights by underscoring the need for equitable access to sequencing technologies and sustained regional collaboration. Such efforts are essential to effectively monitor viral evolution and inform evidence-based interventions in a region historically constrained by limited resources and infrastructure.

This study has several limitations. It relies on publicly available data with limited metadata, assumes that sequenced genomes accurately represent local and regional contexts, and does not account for country-specific factors such as diagnostic and surveillance investments, containment measures, or other relevant country-level indicators. Nonetheless, integrating regional genomic surveillance into national health systems could improve outbreak response times, enhance the detection of emerging variants, and enable more efficient allocation of scarce healthcare resources.

## 5 Conclusions

This overview of SARS-CoV-2 genomic surveillance in Central America and Dominican Republic highlights the critical role of the collaborative efforts between PAHO and COMISCA in enhancing regional sequencing capacity. By improving laboratory resources and fostering cooperation, countries effectively monitored key variants, including Delta and Omicron, demonstrating synchronous transmission dynamics across the region. The rise of recombinant variants emphasizes the ongoing evolution of the virus and the need to sustain genomic monitoring.

This collaborative model provides a robust framework for future public health crises, ensuring readiness to respond to emerging threats. Moving forward, countries should continue to build on these achievements, addressing opportunities for improvement in the sequencing capacity and promoting further collaborations to safeguard public health in the region.

## Data Availability

The original contributions presented in the study are included in the article/Supplementary material, further inquiries can be directed to the corresponding author.
