# Supplementary material for: Overview of SARS-CoV-2 genomic surveillance in Central America and the Dominican Republic from February 2020 to January 2023: the impact of PAHO and COMISCA's collaborative efforts
Source: Front Public Health. 2026 Feb 11;14:1738843. doi: 10.3389/fpubh.2026.1738843 (PMC12932531; doi:10.3389/fpubh.2026.1738843)
Supplement: Supplementary file 3 [file Data_Sheet_1.docx]

Supplementary Material

# Supplementary Figures and Tables

## Supplementary Tables

# Table S1: SARS-CoV-2 genomic surveillance datasets from the GISAID repository, covering Central America and the Dominican Republic from February 2020 to February 2023.

# Table S2: Regression Coefficients from Negative Binomial Models Assessing the Association Between Monthly SARS-CoV-2 Lineage Proportions and Reported Cases and Deaths in Central America and the Dominican Republic, Feb2020–Jan2023.

## Supplementary Figures

**
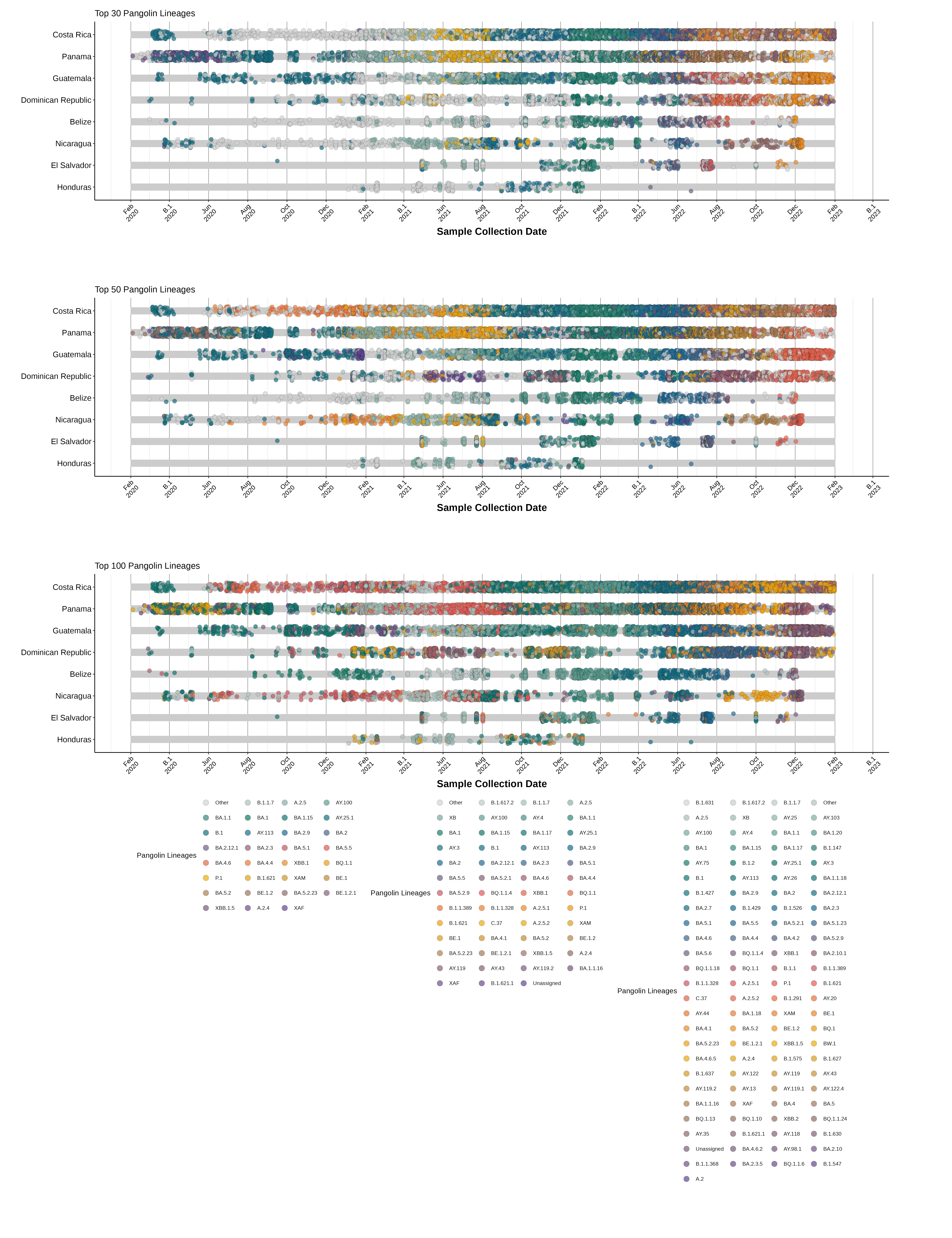
**

**Figure S1.** Complementary Figure 1. Individual SARS-CoV-2 sequences obtained by country in Central America and Dominican Republic from February 2020 to January 2023. Panels show the top 30, 50 and 100 most prevalent lineages individually labelled, all the remaining lineages were labelled as other.
